# Transcriptomic analysis of *Clostridium thermocellum Populus* hydrolysate-tolerant mutant strain shows increased cellular efficiency in response to *Populus* hydrolysate compared to the wild type strain

**DOI:** 10.1186/s12866-014-0215-5

**Published:** 2014-08-16

**Authors:** Jessica L Linville, Miguel Rodriguez, Steven D Brown, Jonathan R Mielenz, Chris D Cox

**Affiliations:** 1 Department of Civil and Environmental Engineering, University of Tennessee, Knoxville, TN, USA; 2 Bioenergy Science Center, Oak Ridge National Laboratory, Oak Ridge, TN, USA; 3 Biosciences Division, Oak Ridge National Laboratory, Oak Ridge, TN, USA; 4 Center for Environmental Biotechnology, University of Tennessee, Knoxville, TN, USA

**Keywords:** Clostridium thermocellum, Populus hydrolysate, Inhibitor tolerance, Gene expression, Transcriptomic, RNA-seq, Consolidated bioprocessing

## Abstract

**Background:**

The thermophilic, anaerobic bacterium, *Clostridium thermocellum* is a model organism for consolidated processing due to its efficient fermentation of cellulose. Constituents of dilute acid pretreatment hydrolysate are known to inhibit *C. thermocellum* and other microorganisms. To evaluate the biological impact of this type of hydrolysate, a transcriptomic analysis of growth in hydrolysate-containing medium was conducted on 17.5% v/v *Populus* hydrolysate-tolerant mutant (PM) and wild type (WT) strains of *C. thermocellum*.

**Results:**

In two levels of *Populus* hydrolysate medium (0% and 10% v/v), the PM showed both gene specific increases and decreases of gene expression compared to the wild-type strain. The PM had increased expression of genes in energy production and conversion, and amino acid transport and metabolism in both standard and 10% v/v *Populus* hydrolysate media. In particular, expression of the histidine metabolism increased up to 100 fold. In contrast, the PM decreased gene expression in cell division and sporulation (standard medium only), cell defense mechanisms, cell envelope, cell motility, and cellulosome in both media. The PM downregulated inorganic ion transport and metabolism in standard medium but upregulated it in the hydrolysate media when compared to the WT. The WT differentially expressed 1072 genes in response to the hydrolysate medium which included increased transcription of cell defense mechanisms, cell motility, and cellulosome, and decreased expression in cell envelope, amino acid transport and metabolism, inorganic ion transport and metabolism, and lipid metabolism, while the PM only differentially expressed 92 genes. The PM tolerates up to 17.5% v/v *Populus* hydrolysate and growth in it elicited 489 genes with differential expression, which included increased expression in energy production and conversion, cellulosome production, and inorganic ion transport and metabolism and decreased expression in transcription and cell defense mechanisms.

**Conclusion:**

These results suggest the mechanisms of tolerance for the *Populus* hydrolysate-tolerant mutant strain of *C. thermocellum* are based on increased cellular efficiency caused apparently by downregulation of non-critical genes and increasing the expression of genes in energy production and conversion rather than tolerance to specific hydrolysate components. The wild type, conversely, responds to hydrolysate media by down-regulating growth genes and up-regulating stress response genes.

## Background

Sugars contained in plant cell walls are a potential form of renewable energy that can be transformed into liquid transportation fuels through fermentation processes. However, the sugars are present in the form of cellulosic and hemicellulosic polymers which prevents direct fermentation of biomass by common industrial microorganisms such as yeast. Cellulose is particularly insoluble and recalcitrant to biodegradation, which represents a major technological hurdle to the realization of a cellulosic biofuels industry. The presence of lignin in the plant cell wall presents additional challenges as it is not easily biodegraded, can limit access to cellulose, and has the potential to form inhibitory byproducts during biomass pretreatment. Certain thermophilic, anaerobic, Gram positive bacteria have shown the ability to biodegrade cellulose and ferment it into ethanol and other fermentation products such as acetate, lactate, formate and hydrogen, giving rise to the possibility of converting cellulose directly to transportation fuels in a single step in a process known as consolidated bioprocessing (CBP). *Clostridium thermocellum* is often considered to be a model organism of this class of bacteria.

Compounds generated during biomass pretreatment, hydrolysis, and microbial fermentation can have inhibitory effects on the fermenting microorganism, which decreases ethanol yields [1],[2] thereby rendering the process uneconomical. Improved tolerance to inhibitory compounds found in pretreated biomass hydrolysate should improve the fermentation process and increase economic feasibility of CBP. Significant clues to the mechanisms involved in adaptation to new environments, such as would be found in a CPB production scheme, have come from studies of gene expression in response to specific stresses [3]. The response of cells to environmental changes can provide clues to the molecular apparatuses that enable cells to adapt to new environments and the molecular mechanisms that have evolved to regulate the remodeling of gene expression that occurs in new environments [3]. By understanding the genetic basis for mechanisms of improved tolerance to inhibitors there is a possibility to rationally engineer their traits in the future [4]–[7].

There have been a number of studies that have analyzed the effect of various stresses associated with biofuel production, product inhibition and inhibitory compounds from pretreated biomass [8]–[14]. Examination of changes in the gene expression profile in response to these stresses can provide mechanistic insight to the physiological response. RNA Sequencing (RNA-seq) is an established technology for quantifying gene expression that has much greater sensitivity and dynamic range than conventional microarray technology [15]. RNA-seq is particularly relevant for controlled experiments comparing the expression in wild type and mutant strains of an organism [16]. Moreover, combining RNA-seq with genomic data can help identify genetic loci responsible for variation in gene expression between individuals [16].

The development of a *Populus* hydrolysate tolerant strain of *C. thermocellum,* which grows as well in 17.5% v/v *Populus* hydrolysate as the wild type (WT) does in standard medium, has been reported [17]. Genomic analysis of the mutant strain (termed PM for *Populus* mutant) revealed several mutations in the strain that may be responsible for its faster growth rate and tolerance to *Populus* hydrolysate with selected mutations related to the transcriptional changes [17]. The extent of the growth, end product production and *Populus* hydrolysate tolerance was described by kinetic modeling [18]. In the present study, the WT and PM strains were grown in various concentrations of *Populus* hydrolysate (0% or standard medium, 10% and 17.5% v/v *Populus* hydrolysate) and a genome-wide transcriptomic analysis was conducted at mid-log and late-log time points via RNA-seq. In addition to changes in transcription levels, post-transcriptional regulation of gene expression through the action of sRNA molecules has been demonstrated to play a key role in stress response in Clostridia [19]; however, the focus of this paper is on changes in gene regulation at the transcriptional level. Two types of comparisons were used to further elucidate the potential mechanism(s) of tolerance for the PM strain: a comparison of the strains in standard and hydrolysate media and a comparison of each strain’s response to *Populus* hydrolysate-containing media using its gene expression profile in standard medium as a baseline.

## Results

### Fermentative growth

Batch fermentations were conducted for the *Populus* mutant (PM) and wild type (WT) strains of *C. thermocellum* as previously reported in Linville et al. [17]. Samples were taken at regular intervals from each fermentation unit based on their growth rate and analyzed for optical density (OD_600_) and metabolite concentration by HPLC. The dry cell weight (DCW) of the samples was determined by calibration curve (data not shown). In brief, the PM had approximately twice the growth rate when compared to the WT in standard medium [17],[18]. The PM also produced 1.1-1.3 times more ethanol and the same amount of acetic acid than the WT under the same test conditions [17],[18]. The dry cell weight, sugar utilization, ethanol production and acetic acid production for the fermentations are shown in (Additional file 1: Figure S1). The samples for RNA analysis were harvested from the fermentors during the mid-log and late-log phase. The time points and dry cell weight of the mid-log and late-log phase can be seen in (Additional file 1: Table S1).

### RNA-seq analysis

An analysis of variance (ANOVA) was conducted on each of the independent variables separately: strain, *Populus* hydrolysate concentration, and time. Differentially expressed genes were defined as a 2-fold change in expression with a false discovery rate of less than 5% (p < 0.05). Of the 3,236 genes in *C. thermocellum*, roughly 18% (n = 574) showed a difference in expression between strains. Furthermore, approximately 16% (n = 505) of the genes showed a change in expression between the three concentration comparisons. None of the genes showed a change in expression between the two time points. Since, there were no statistically significant changes in expression of individual genes between the mid-log and late-log time points, the analysis considered-between strain and between-hydrolysate-concentration comparisons to be significantly different if the expression differences were significant for either of these two time points.

**Figure 1:**
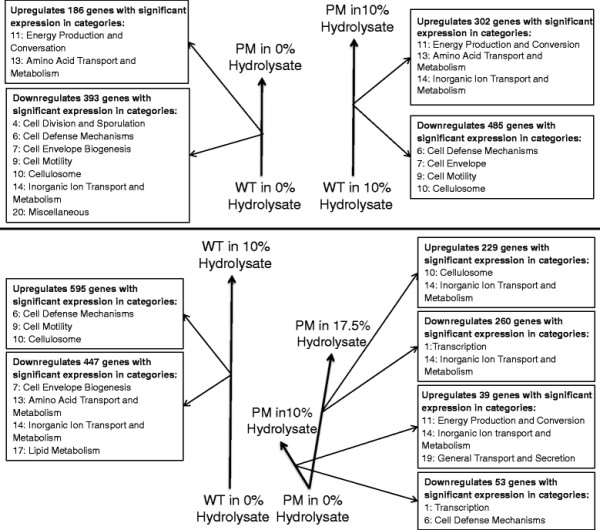
**Pictorial representation of the four gene expression comparisons.** The top half of the graph shows the strain comparison and the bottom half shows the hydrolysate media comparison. Heavy black arrows indicate the direction of comparison for transcriptomic analysis. Length of the arrow is used to indicate number of differentially expressed genes. The condition at the base of the arrow was used as the baseline of the comparison. Thin black arrows point to boxes that list the number of statistically significant up- or-down regulated genes and the categories with significant changes in expression in that direction.

Simple comparisons only consider the differences in gene expression from changing one of the three variables at a time: strain, *Populus* hydrolysate concentration or time. The ANOVA of the three independent variables in combination revealed approximately 55% (n = 1795) of the genes were differentially expressed in at least one of the simple comparisons (Additional file 2). Two types of analyses are the focus of this paper. The first analysis compares gene expression in the WT and PM strains in 0% v/v and 10% v/v *Populus* hydrolysate. A positive differential expression (upregulation) represents a higher expression level in the PM strain and a negative differential expression (downregulation) represents a lower expression level in the PM strain when compared to the WT strain. The second type of analysis compares gene expression under different concentrations of *Populus* hydrolysate within a given strain as follows: the PM in 0% versus 10% v/v *Populus* hydrolysate and 0% versus 17.5% v/v *Populus* hydrolysate, and the WT in 0% versus 10% v/v *Populus* hydrolysate. For these comparisons a positive differential expression (upregulation) represents an increase in expression level and a negative differential expression (downregulation) represents a decrease in expression level in the *Populus* hydrolysate compared to standard medium. Of the 1795 differentially expressed genes, 1740 are represented by these four comparisons. The remaining 55 genes are differentially expressed between the comparisons of the PM in 10% versus 17.5% v/v *Populus* hydrolysate or between the mid-log versus late-late log time points for a given condition. Genes that encode for proteins classified as hypothetical, uncharacterized or unknown function, accounting for 551 of the 1740 genes, were removed from further analysis (Additional file 3). The remaining 1189 differentially expressed genes were then assigned to one of 20 categories based on function (Additional file 4). To determine if genes within a given category were systematically regulated, the statistical significance of the odds ratio of the number of up- or down-regulated genes within a category versus the total number of up- or down- regulated genes in *C. thermocellum* was calculated. This process is similar to the categorical analysis of other clostridia species [12]–[14]. Lists of the total and differentially expressed genes by category and the total number of differentially expressed genes for each analysis are provided (Additional file 1: Table S2). Figure 1 is a pictorial representation of the five comparisons indicating the total number (including hypothetical genes) of differentially expressed genes and the categories with significant change in expression as determined by odds ratio.

Changes in gene expression level as determined by RNA-seq were confirmed using real-time quantitative PCR (qPCR) for six genes from the WT versus PM in 0% v/v *Populus* hydrolysate mid-log comparison (Additional file 1: Figure S2). The coefficient of determination R^2^ = 0.92 was obtained for comparisons of gene expression as determined by RNA-seq and qPCR (Additional file 1: Figure S2), which indicated RNA-seq data was of good quality.

## Discussion

### Strain comparison

The strain comparison analyzes the difference in expressed genes between the WT and PM in standard and hydrolysate media to elucidate the effect of the mutations. The 186 upregulated genes versus the 393 downregulated genes in standard medium and the 371 upregulated genes versus the 780 downregulated genes in 10% v/v *Populus* hydrolysate medium for the PM compared to the WT supports the hypothesis that the PM appears to have a more efficient cellular metabolism due to more downregulated gene expression, which leads to increased robustness regardless of the growth conditions (Figure 1). For example, PM grows at twice the rate of the WT in standard medium, indicating its greater metabolism capability or “robustness” [18]. The *Populus* hydrolysate tolerant phenotype of the PM is the result of two simultaneous mechanisms of action: increases in cellular repair and altered energy metabolism [17]. These mutations not only alter tolerance to the *Populus* hydrolysate but also alter the growth rate in standard medium suggesting a more global change in gene expression which will be evaluated by comparing the change in expression between the PM and WT strain in both standard (0% v/v *Populus* hydrolysate) and *Populus* hydrolysate media.

The PM has a non-synonymous single nucleotide polymorphism (SNP) in a strongly conserved amino acid of the single copy of the *rpoB* gene (Cthe_2724, E885K) which encodes for a DNA directed RNA polymerase, beta subunit [17]. The beta subunit of the RNA polymerase interacts directly with both the DNA and has weak binding sites for the sigma factor [20]. This mutation potentially changes the specificity, activity and/or stability of the RNA polymerase which has the potential to affect a large number of genes through the promoter interaction [17],[21]–[23]. In addition, mutations in *rpoB* have been shown to block the uptake of aromatic compounds by the membrane transport system therefore, increasing tolerance [24]. The PM differentially expresses multiple sigma factors when compared to the WT in standard medium which can be directly linked to the overall change in expression for certain categories of genes. The differentially expressed sigma factors are listed in Table 1 and will be discussed in the context of the genes they regulate.

**Table 1:** Fold change in expression of sigma factors

**Gene name**	**Product**	**PM vs. WT 0**		**PM vs. WT 10**		**PM 0 vs. 10**		**PM 0 vs. 17.5**		**WT 0 vs. 10**	
		**ML**	**LL**	**ML**	**LL**	**ML**	**LL**	**ML**	**LL**	**ML**	**LL**
Cthe_1272	sigma-70 region 2 domain protein	2.34	1.24	**−5.64**	**−3.59**	−2.20	−1.64	−1.38	1.94	**6.00**	**2.72**
Cthe_0195	Sigma-70 region 4 type 2	**2.80**	1.61	−2.48	−1.42	−2.06	−1.23	−1.44	1.49	**3.37**	**1.86**
Cthe_1438	RNA polymerase sigma factor, sigma-70 family	**2.68**	**2.06**	1.70	−1.38	**−2.26**	−1.76	**−2.95**	**−2.42**	−1.43	1.61
Cthe_0890	RNA polymerase sigma factor, sigma-70 family	−1.09	−1.63	**−2.01**	−1.12	1.45	−1.64	−1.27	−1.14	−1.13	1.21
Cthe_1809	RNA polymerase sigma factor, sigma-70 family	**18.26**	**16.44**	**24.37**	**13.05**	−1.69	**−2.11**	**−4.55**	**−4.06**	**−2.25**	−1.68
Cthe_0446	sigma-E processing peptidase SpoIIGA	−1.86	**−2.21**	−1.14	1.26	−1.10	1.45	−1.03	1.51	−1.78	−1.92
Cthe_0447	RNA polymerase sigma-E factor	1.90	**2.58**	**2.15**	1.91	−1.56	−1.19	−1.30	**−2.65**	−1.77	1.14
Cthe_0120	RNA polymerase sigma-F factor	1.71	**2.01**	**2.48**	1.96	1.01	1.15	−1.03	−1.22	−1.43	1.18
Cthe_0448	RNA polymerase sigma-G factor	−1.79	−2.55	1.09	−1.14	−2.10	−1.23	−1.56	−1.06	**−4.11**	**−2.73**
Cthe_1012	RNA polymerase sigma-K factor	**−3.94**	**−4.74**	**−2.88**	**−2.96**	1.13	1.20	1.07	**3.57**	−1.21	−1.33
Cthe_2059	RNA polymerase sigma-H factor	1.45	1.65	1.86	1.03	−1.30	−1.52	−1.41	**−2.13**	−1.66	1.05
Cthe_0074	RNA polymerase, sigma-24 subunit, ECF subfamily	−1.19	−1.46	−1.87	**−2.22**	3.64	1.40	3.54	1.74	**5.71**	**2.13**
Cthe_0495	RNA polymerase, sigma 28 subunit	**−3.04**	**−3.47**	**−9.98**	**−4.44**	1.18	1.43	1.37	1.53	**3.87**	**1.83**
Cthe_2100	transcriptional regulator, AbrB family	2.21	2.48	**8.86**	1.29	−2.67	−1.16	**−5.28**	**−13.66**	**−10.68**	**1.66**
Cthe_0315	RNA polymerase sigma-I factor	−1.40	−2.19	**−4.00**	**−3.49**	−1.13	−1.17	−1.00	1.92	2.52	1.36
Cthe_2975	RNA polymerase sigma-I factor	1.24	1.47	**−7.26**	**−2.59**	−2.09	−1.94	1.15	1.45	4.32	1.96
Cthe_0403	RNA polymerase sigma-I factor	−1.65	−1.92	**−5.17**	**−3.64**	1.89	1.76	−1.66	−1.08	−1.12	1.83

Bold values indicate significantly different expression levels as determined by ANOVA. For the PM vs. WT in 0% and 10% v/v *Populus* hydrolysate a positive/negative value represents a higher/lower level of expression in the PM compared to the WT. For the standard medium (0%) versus *Populus* hydrolysate media (10 or 17.5%) a positive/negative value represents a higher/lower expression in the hydrolysate media compared to standard medium. Values are indicated for samples collected during the mid-log (ML) and late-log (LL) growth phases.

#### Categories of gene with increased expression in the PM

The PM increases the gene expression in only two categories compared to the WT in standard and *Populus* hydrolysate media: energy production and conversion, and amino acid transport and metabolism (Figure 1). In addition to these, the PM also increases the expression of inorganic ion metabolism and transport genes compared to the WT in 10% v/v *Populus* hydrolysate medium. The increased expression in the energy production and conversion genes may allow for the increased growth phenotype observed in the PM strain. Increases in glycolysis would lead to increases in reducing power (in the form of NADH) being available for downstream electron transport and ethanol production. The increase in ethanol production and increase in electron flux may generate sufficient NAD^+^ to ensure increased cellular metabolism [8].

**Figure 2:**
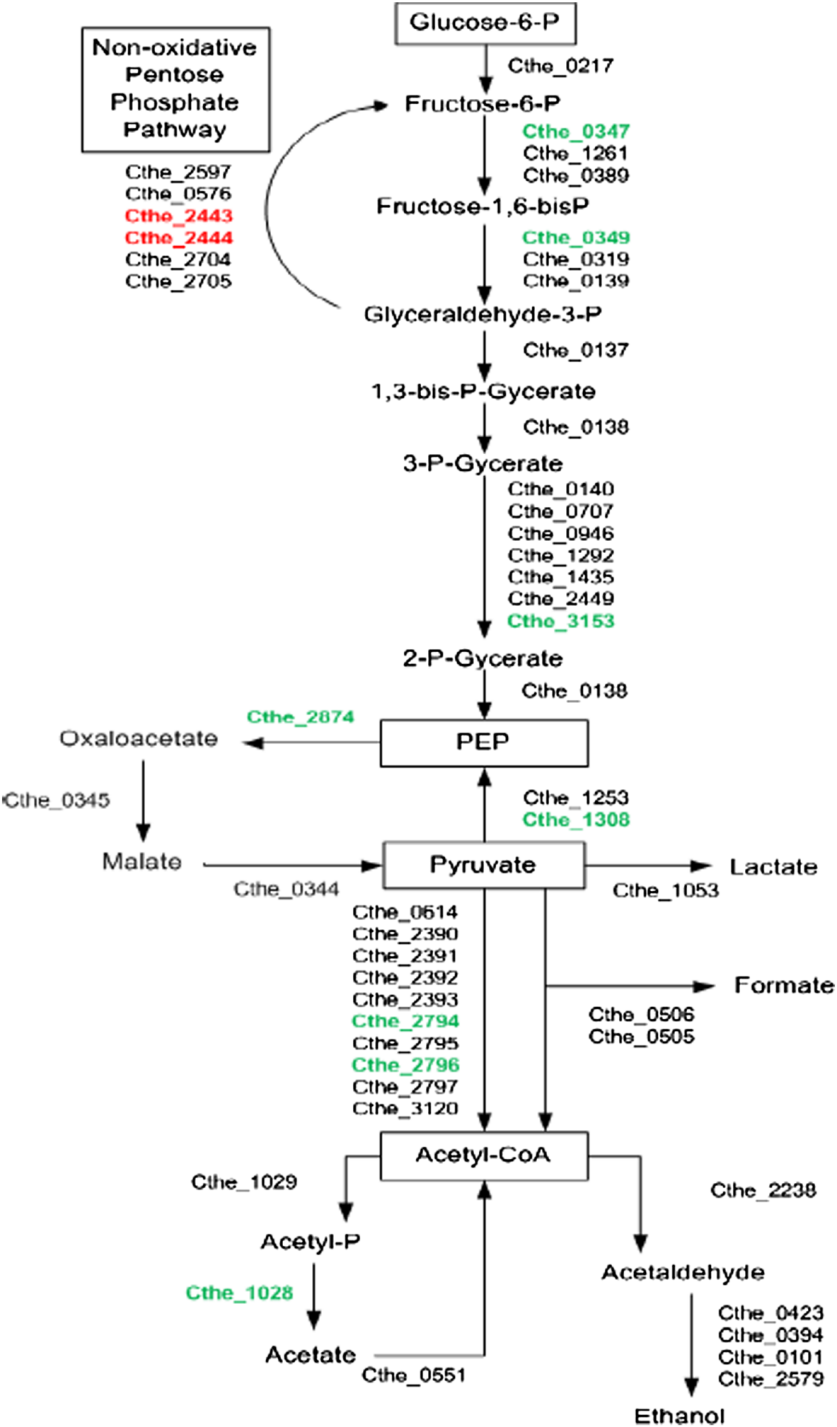
**Central metabolism of*C. thermocellum*with differentially expressed genes between the WT and PM higlighted.** Genes colored geen have greater than 2-fold higher expression and genes colored red have a greater than 2-fold lower expression in the PM than the WT in standard media. The extent of gene expression change and expression levels for the other comparisons are given in Table 2.

The assemblage of genes encoding proteins involved in pyruvate metabolism and end-product synthesis dictate, in part, how carbon and electrons flux is distributed between the catabolic, anabolic, and energy producing pathways of the cell [25]. *C. thermocellum* catabolizes glucose via the Embden-Meyerhof pathway using the “malate shunt” (Figure 2) [26]–[28]. Compared to the WT, the PM had a higher expression of 23 and 44 genes belonging to the energy production and conversion category in standard and *Populus* hydrolysate media, respectively. The PM upregulated 8 genes specific to the central metabolism and mixed-acid fermentation compared to the WT in standard medium (Figure 2 and Table 2). In 10% v/v *Populus* hydrolysate medium, the PM upregulated 10 genes along the central metabolism and mixed acid fermentation pathways compared to the WT. The PM has a mutation in the non-coding region upstream of the Cthe_0422-Cthe_0423 operon which encodes the *rex* (redox) repressor and the *adhE* alcohol dehydrogenase. This mutation may cause the observed increase in ethanol production [17],[18]. A study of the effect of cellulose fermentation found that the central metabolism genes are typically upregulated during cellulose fermentation compared to cellobiose fermentation that the cells were grown on in this study [12],[25]. The native upregulation of these genes by the PM may allow for the phenotypically faster growth rate.

**Table 2:** Fold change in gene expression along the central metabolism and mixed-acid fermentation pathways

**Gene**	**Product**	**PM vs. WT 0**		**PM vs. WT 10**		**PM 0 vs. 10**		**PM 0 vs. 17.5**		**WT 0 vs. 10**	
		**ML**	**LL**	**ML**	**LL**	**ML**	**LL**	**ML**	**LL**	**ML**	**LL**
glucose-6-phosphate to PEP											
Cthe_0347	Phosphofructokinase	1.77	**2.59**	**2.97**	**2.13**	−1.35	−1.31	−1.14	−1.49	**−2.27**	−1.07
Cthe_0349	fructose-1,6-bisphosphate aldolase, class II	1.60	**2.49**	**3.31**	**2.50**	−1.52	−1.41	−1.49	**−2.03**	**−3.14**	−1.42
Cthe_2449	Phosphoglycerate mutase	−2.46	−1.85	1.42	−1.74	−1.48	−1.90	−2.01	−2.88	**−5.18**	**−2.03**
Cthe_3153	alpha-ribazole phosphatase	**2.11**	**2.33**	1.40	1.40	1.21	1.23	1.42	−1.14	1.82	**2.04**
Cthe_0143	Enolase	−1.23	−1.04	1.63	−1.02	−1.11	−1.13	−1.05	**−2.96**	**−2.22**	−1.16
Non-oxidative Pentose Phosphate pathway											
Cthe_2443	Transketolase domain-containing protein	**−3.24**	**−4.70**	1.02	−3.00	1.14	−1.75	−1.90	−1.52	−2.88	−2.74
Cthe_2444	Transketolase domain-containing protein	**−3.47**	**−4.63**	−1.15	−3.39	1.26	−1.72	−1.63	−1.57	−2.41	−2.36
Cthe_2705	Transketolase central region	−1.44	−1.33	**2.25**	1.19	1.24	1.21	1.17	−1.81	**−2.60**	−1.32
PEP to Pyruvate											
Cthe_2874	Phosphoenolpyruvate carboxykinase [GTP]	1.39	**2.46**	**1.43**	**2.09**	−1.05	−1.04	1.30	1.38	−1.07	1.14
Cthe_0344	malic protein NAD-binding	−1.68	1.06	1.26	−1.10	−1.01	−1.14	1.20	−1.27	**−2.13**	1.02
Cthe_1308	pyruvate, phosphate dikinase	1.64	**2.29**	−1.30	1.65	−1.05	1.07	1.30	1.10	**2.03**	1.49
Pyruvate to Lactate/Formate/Acetyl-CoA											
Cthe_1053	L-lactate dehydrogenase	−1.78	−1.25	1.32	−1.02	−1.41	−1.27	−1.33	−1.16	**−3.30**	−1.55
Cthe_2794	pyruvate/ketoisovalerate oxidoreductase, gamma subunit	**4.30**	1.48	**5.15**	**3.99**	1.92	2.56	2.45	2.78	1.61	−1.05
Cthe_2796	pyruvate flavodoxin/ferredoxin oxidoreductase domain protein	**3.13**	1.47	**4.16**	**2.94**	1.98	2.05	1.88	1.94	1.49	1.02
Cthe_0505	formate acetyltransferase	−1.95	−1.91	1.46	−1.04	1.24	1.04	1.11	−1.81	**−2.31**	−1.76
Acetyl-CoA to Ethanol/Acetate											
Cthe_1028	Acetate kinase	1.67	**2.57**	**3.63**	**3.05**	**2.12**	1.26	**2.76**	1.50	−1.02	1.06
Cthe_1029	phosphate acetyltransferase	1.54	1.79	**4.01**	**3.83**	**2.42**	1.33	**2.73**	1.63	−1.08	−1.61
Cthe_2238	Aldehyde Dehydrogenase	1.06	1.04	−1.81	−1.29	1.20	1.36	1.36	1.02	**2.30**	1.83
Cthe_0101	iron-containing alcohol dehydrogenase	−1.35	−1.19	**2.19**	1.20	1.22	−1.04	−1.18	−1.82	**−2.43**	−1.48
Cthe_0423	iron-containing alcohol dehydrogenase	1.12	1.07	**4.75**	**5.02**	1.26	1.06	1.28	1.45	**−3.36**	**−4.42**

Bold values indicate significantly different levels of expression as determined by ANOVA. For the PM vs. WT in 0% and 10% v/v *Populus* hydrolysate, a positive/negative value represents a higher/lower expression level in the PM compared to the WT. For the standard medium (0%) versus *Populus* hydrolysate media (10 or 17.5%) positive/negative values represents higher/lower expression levels in the hydrolysate media compared to standard medium. Values are indicated for samples collected during mid-log (ML) and late-log (LL) growth phases.

*C. thermocellum* uses the hydrogenase-mediated pathway for production of molecular hydrogen to dispose the excess reducing equivalents generated during carbohydrate catabolism [12],[28]. In the process, the Ech hydrogenase complex pump H^+^/Na^+^ ions across the cell membrane and create proton gradients for powering ATP synthesis by ATP synthase (ATPase) [12]. The PM has a mutation in the non-coding region 127 bp upstream of the F-type ATP synthase operon (Cthe_2602 – Cthe_2609) which may lead to an increase in the expression of this gene cluster in the PM compared to the WT in standard medium (Table 3) [17]. The PM also increases the expression of 4 and 8 genes in the Ech hydrogenase complex (Cthe_3013-3024) compared to the WT in standard and *Populus* hydrolysate media (Table 3). The effect of the increased expression of the ATPase and Ech-type hydrogenases on the electron flux in the cell is unknown at the time [17]. However, analysis of the H_2_ production rate of PM and WT in 0% and 10% v/v *Populus* hydrolysate media shows no significant difference [17]. In addition, regardless of the strain or growth medium, the five other hydrogen producing complexes in *C. thermocellum* are expressed at levels between 4 and 50 times greater than the Ech-type hydrogenases (data not shown) [12]. Collectively these results argue against the increased activity of Ech-type hydrogenase complex significantly changing the electron flux in the PM. Another possibility for this change in gene expression could be electron bifurcation which was recently found in anaerobic microbes. For example, *Acetobacterium woodii* employs a sodium-motive ferredoxin: NAD^+^-oxidoreductase (Rnf complex) that couples the exergonic electron flow from reduced ferredoxin to NAD^+^ to establish a transmembrane electrochemical Na^+^ gradient that then drives the synthesis of ATP via a well characterized Na^+^ F_1_F_0_- ATP synthase [29]. The data showed that the complex was reduced by the [FeFe]- hydrogenase of *A. woodii* and reduction of one was strictly dependent on the presence of the other electron acceptor [29]. *Clostridium kluyveri* have also been shown to catalyze acetyl-CoA and ferredoxin-dependent formation of H_2_ from NADH [30].

**Table 3:** Fold change in gene expression involved in cellular redox

		**PM vs. WT 0**		**PM vs. WT 10**		**PM 0 vs. 10**		**PM 0 vs. 17.5**		**WT 0 vs. 10**	
		**ML**	**LL**	**ML**	**LL**	**ML**	**LL**	**ML**	**LL**	**ML**	**LL**
Redox transcriptional repressor											
Cthe_0422	Redox-sensing transcriptional repressor rex	1.13	−1.08	**7.01**	**5.53**	1.04	−1.02	−1.04	−1.11	**−5.96**	**−6.08**
Ech-type hydrogenases											
Cthe_3013	hydrogenase expression/formation protein HypE	1.39	1.19	**3.42**	**2.34**	−1.90	−2.24	1.30	−1.14	1.37	−1.03
Cthe_3016	[NiFe] hydrogenase maturation protein HypF	2.34	2.42	**3.10**	**3.62**	−1.03	−1.45	1.28	1.03	1.52	1.84
Cthe_3017	hydrogenase accessory protein HypB	2.66	3.07	**2.56**	**3.73**	1.12	−1.15	1.08	1.06	1.63	1.97
Cthe_3018	hydrogenase expression/synthesis HypA	2.51	3.11	**2.20**	**3.99**	1.40	−1.07	1.22	1.20	1.92	2.27
Cthe_3019	4Fe-4S ferredoxin iron-sulfur binding domain-containing protein	**2.89**	**3.12**	**1.77**	**2.98**	1.14	1.03	1.54	**2.02**	1.87	1.08
Cthe_3020	NADH-ubiquinone oxidoreductase chain 49 kDa	**2.96**	**3.83**	**1.86**	**3.15**	1.02	−1.03	1.55	**2.07**	1.64	1.18
Cthe_3021	ech hydrogenase, subunit EchD, putative	**4.29**	**4.79**	**2.15**	**3.03**	−1.12	−1.09	1.16	1.71	1.79	1.46
Cthe_3024	NADH/Ubiquinone/plastoquinone (complex I)	**2.04**	**2.26**	**2.83**	**2.10**	−1.12	−1.10	−1.17	1.05	−1.54	−1.02
ATP synthase											
Cthe_2602	ATP synthase subunit a	**2.77**	**3.55**	**5.58**	**3.87**	−1.21	1.10	−1.35	−1.72	**−2.44**	1.01
Cthe_2603	ATP synthase subunit c	**2.27**	**2.68**	**2.66**	**4.62**	1.11	1.04	−1.04	−1.22	−1.06	−1.66
Cthe_2604	ATP synthase subunit b	**2.48**	**2.18**	**4.03**	**4.30**	−1.01	1.10	−1.23	−1.32	−1.64	−1.80
Cthe_2605	ATP synthase F1, delta subunit	**3.55**	**2.04**	**3.86**	**2.95**	−1.06	−1.05	−1.77	**−2.01**	−1.15	−1.53
Cthe_2606	ATP synthase F1, alpha subunit	**2.40**	**2.00**	**2.75**	**3.27**	1.60	1.31	1.20	1.25	1.40	−1.24
Cthe_2607	ATP synthase F1, gamma subunit	**2.63**	**2.09**	**2.06**	**2.95**	1.17	1.20	1.09	1.49	1.50	−1.18
Cthe_2608	ATP synthase F1, beta subunit	**2.67**	**2.65**	**3.73**	**4.36**	1.40	1.37	1.04	1.05	−1.00	−1.20
Cthe_2609	ATP synthase epsilon chain	**2.94**	**2.87**	**4.11**	**4.79**	1.12	1.33	−1.21	−1.11	−1.24	−1.26

Bold values indicate significantly different levels of expression as determined by ANOVA. For the PM vs. WT in 0% and 10% v/v *Populus* hydrolysate, a positive/negative value represents a higher/lower expression level in the PM compared to the WT. For the standard medium (0%) versus *Populus* hydrolysate media (10 or 17.5%) positive/negative values represents higher/lower expression levels in the hydrolysate media compared to standard medium. Values are indicated for samples collected during mid-log (ML) and late-log (LL) growth phases.

Furthermore, sigma factor σ^A^ is the principle sigma factor present in vegetatively growing *B. subtilis* and other Gram-positive bacteria [31] and it directs transcription of genes important to metabolism [23]. There are 10 genes that encode for σ^A^ subunits in *C. thermocellum*. Three of the genes that encode for σ^A^ (Cthe_0195, Cthe_1438 and Cthe_1809) are upregulated in the PM compared to the WT in standard conditions (Table 1). The change in expression of these three sigma factors were considered significant based on the subset odds ratio of the total number of σ^A^. Oddly, the PM has a lower expression of two genes that encode for σ^A^ (Cthe_0890, and Cthe_1272) in 10% v/v *Populus* hydrolysate compared to the WT; however, the PM does still increase the expression of Cthe_1809. Cthe_1809 had 18-fold greater expression level at the mid-log time point in standard media and 24-fold higher expression level at the mid-long time point in 10% v/v *Populus* hydrolysate for the PM versus WT. The higher expression level may contribute to the higher observed growth rate phenotype and energy production/conservation in the PM strain under standard conditions [17],[18].

**Figure 3:**
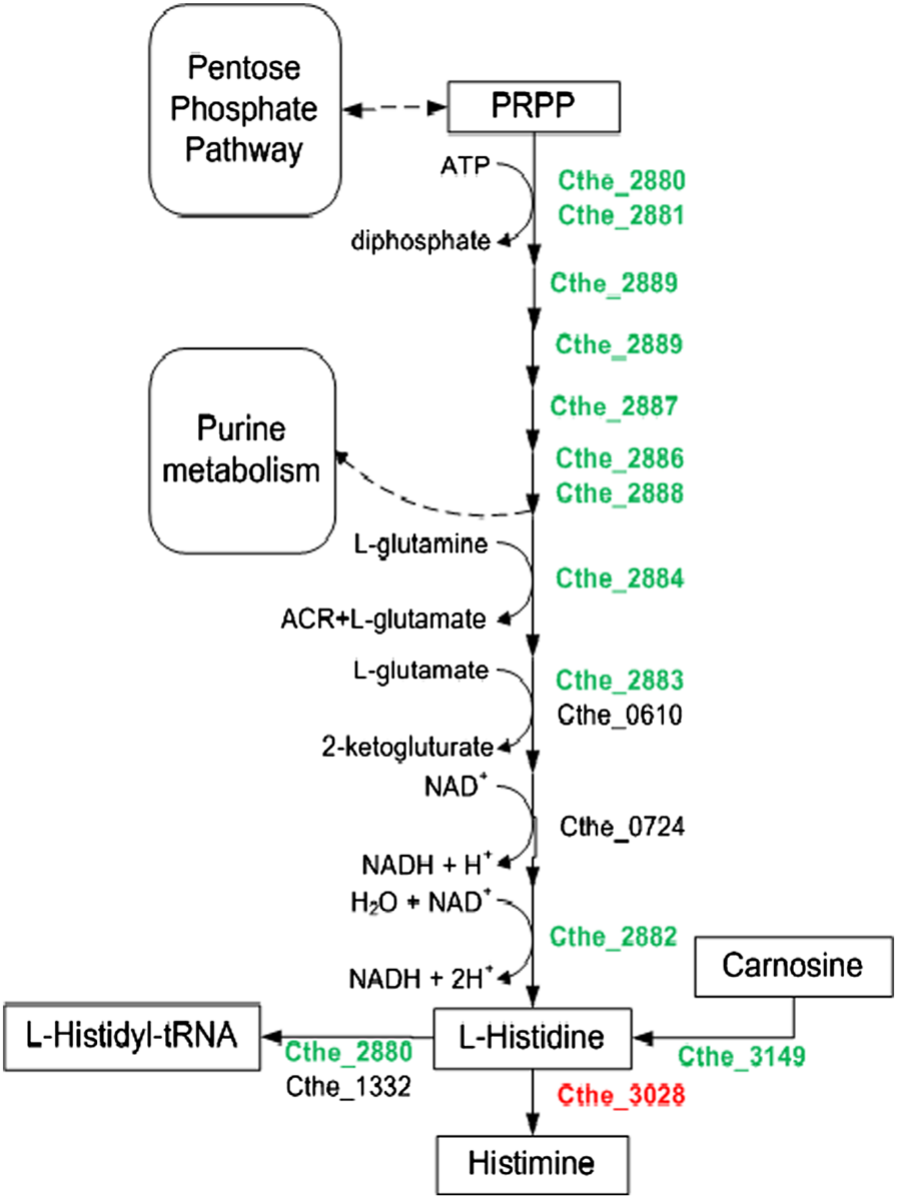
**The PM has increased expression of genes in the hisidine biosynthesis pathway compared to the WT in standard media.** Genes colored geen have greater than 2-fold higher expression and genes colored red have a greater than 2-fold lower expression in the PM than the WT in standard media. The extent of gene expression change and expression levels in other comparisons are given in Table 4. PRPP, 5-phosphoribosyl 1-pyrophosphate. ACR, aminoimidazole carboxamide ribonucleotide.

Of the 163 genes that encode for various parts of the amino acid transport and metabolism, the PM upregulated a significant number of genes (20 and 37 genes) compared to the WT in standard and *Populus* hydrolysate media. Most significantly, the PM increased the expression of 10 of the 15 genes along the histidine metabolism pathway compared to the WT in standard medium (Table 4). Cthe_2880-Cthe_2889 is a single operon and is among the most highly differentially expressed genes in the PM versus WT comparison, with an average 23-fold to 31-fold increase in expression in standard and *Populus* hydrolysate media. The PM decreases the expression of one gene in this pathway, Cthe_3028 which converts histidine to histamine (Figure 3). De novo biosynthesis of histidine during fermentation may be constrained by the high NADH/NAD^+^ ratio during anaerobic growth and the requirement for further reduction of NAD^+^ in the two terminal steps of biosynthesis [17]. Histidine may be limited by the addition of furfural [17]. The PM has two mutations involved with glutamate catabolism; a possible gain in function in *argD* (Cthe_1866, E55G) and a possible loss in function in *proB* (Cthe_1766, A149T) [17]. These two mutations seem to be a beneficial shift from proline production to glutamate and arginine production in PM [17],[18],[32]. The shift in amino acid production may also assist in the increased expression in the histidine pathway since glutamate is utilized in the pathway. The PM also significantly increases the expression of 6 of the 18 genes belonging to valine, leucine and isoleucine biosynthesis, which may help balance carbon and electron flow. An increase in amino acid production can also help overcome weak acid stress [17],[18],[33].

**Table 4:** Fold change in gene expression in histidine metabolism pathways

**Gene**	**Product**	**PM vs. WT 0**		**PM vs. WT 10**		**PM 0 vs. 10**		**PM 0 vs. 17.5**		**WT 0 vs. 10**	
		**ML**	**LL**	**ML**	**LL**	**ML**	**LL**	**ML**	**LL**	**ML**	**LL**
Cthe_2880	ATP phosphoribosyltransferase regulatory subunit	**98.42**	**29.12**	**98.72**	**80.51**	1.25	1.01	1.12	−1.06	1.25	**−2.73**
Cthe_2881	ATP phosphoribosyltransferase	**78.48**	**23.79**	**85.06**	**100.15**	1.64	1.24	1.35	−1.01	1.52	**−3.40**
Cthe_2882	histidinol dehydrogenase	**35.86**	**18.44**	**28.45**	**44.69**	1.49	1.33	1.37	1.40	1.88	−1.83
Cthe_2883	histidinol-phosphate aminotransferase	**38.12**	**19.61**	**23.12**	**40.22**	1.15	1.22	1.19	1.42	1.89	−1.69
Cthe_2884	Imidazoleglycerol-phosphate dehydratase	**7.45**	**7.71**	**17.31**	**17.09**	1.23	1.25	1.18	1.27	−1.89	−1.77
Cthe_2886	Imidazole glycerol phosphate synthase subunit hisH	**11.99**	**12.29**	**14.84**	**15.87**	1.19	1.12	1.09	−1.01	−1.04	−1.16
Cthe_2887	1-(5-phosphoribosyl)-5-[(5-phosphoribosylamino)methylideneamino] imidazole-4-carboxamide isomerase	**13.46**	**11.01**	**10.02**	**14.54**	1.44	1.20	1.29	1.13	1.93	−1.10
Cthe_2888	Imidazole glycerol phosphate synthase subunit hisF	**12.46**	**14.23**	**10.04**	**18.19**	1.61	1.30	1.54	1.24	1.99	1.02
Cthe_2889	Histidine biosynthesis bifunctional protein hisIE	**12.71**	**10.80**	**6.09**	**12.49**	1.48	1.29	1.51	1.28	**3.08**	1.11
Cthe_3028	Pyridoxal-dependent decarboxylase	**−11.35**	**−13.46**	**−7.10**	**−6.92**	−2.37	−1.04	**−3.78**	**−2.89**	**−3.79**	**−2.02**
Cthe_3149	aminoacyl-histidine dipeptidase	**3.34**	**4.23**	−1.07	1.63	1.15	1.05	1.39	1.37	**4.09**	**2.72**
Cthe_1332	Histidyl-tRNA synthetase	−1.58	−1.89	1.66	−1.18	1.10	−1.03	−1.15	−1.62	**−2.38**	−1.64

Bold values indicate significantly different levels of expression as determined by ANOVA. For the PM vs. WT in 0% and 10% v/v *Populus* hydrolysate, a positive/negative value represents a higher/lower expression level in the PM compared to the WT. For the standard medium (0%) versus *Populus* hydrolysate media (10 or 17.5%) positive/negative values represents higher/lower expression levels in the hydrolysate media compared to standard medium. Values are indicated for samples collected during mid-log (ML) and late-log (LL) growth phases.

#### Categories of gene with decreased expression in the PM

There are a number of categories with decreased expression level for the PM when compared to the WT in standard medium. The downregulation of these genes may be a result of trying to conserve cellular resources and redirect them in such a way as to increase the growth rate for the PM. The downregulated categories will be discussed briefly below.

The downregulation of the cell division and sporulation genes by the PM compared to the WT in standard medium may seem counterintuitive with the faster growth rate of the PM. However, the genes in this category can be subdivided into cell division genes and sporulation genes. Independent odds ratios on the gene subsets show that only the sporulation genes were significantly downregulated by the PM in standard medium (Additional file 1: Table S3). Although the PM downregulates a greater number (23 compared to 20) of cell division and sporulation genes in the 10% v/v *Populus* hydrolysate medium comparison over standard medium, it is not considered significant by odds ratio due to the larger total number of genes that were down regulated in the 10% v/v *Populus* hydrolysate medium comparison. Similarly, the PM downregulates 17 genes belonging to the sporulation subcategory, however, it is not significant in the hydrolysate medium comparison as seen in Additional file 1: Table S3. There are two possible reasons that the PM downregulates the sporulation genes.

The first possible reason for the reduction in sporulation genes is a stop codon placed at the 76 amino acid in the coding region of a distantly related *spo0A* homologue (Cthe_3087) in the PM which should disrupt the gene function; although, the gene expression does not change significantly [17]. There is a second copy of *spo0A* in *C. thermocellum*, Cthe_0812 which is significantly downregulated by an unknown mechanism in standard conditions compared to the WT. The *spo0A* protein is activated when phosphorylated and has been shown to regulate sporulation in a number of clostridia [34]. Although, it is rare for *C. thermocellum* to go into sporulation, it has been shown that sporulation will occur under vitamin limitation, oxygen stress and switching between soluble and insoluble substrates [35]. The PM growth kinetics is consistent with other *spo0A* defective mutants which continue to grow under nutrient limiting conditions [36]–[39].

The second reason for a reduction in the expression of sporulation genes may be that the PM differentially expresses the sigma factors that control sporulation. The five known sporulation sigma factors in *B. subtilis* are σ^E^, σ^F^, σ^G^, σ^H^ and σ^K^[31],[34]. In *B. subtilis*, σ^H^ is the earliest sporulation sigma factor [34]. σ^E^ is the mother cell-specific sigma factor and is also involved in the synthesis of σ^K^, the late-acting mother cell sigma factor [31]. Furthermore, σ^F^ – dependent transcription appears to be limited to the early expression of forespore-specific genes and σ^G^ appears to encode products that are synthesized within the forespore compartment during the later stages of sporulation to enhance spore survival and facilitate germination [31]. There are six genes that encode the various sporulation sigma factors in *C. thermocellum*. The PM has increased expression in σ^E^ (Cthe_0447) and σ^F^ (Cthe_0120), and decreased expression in σ^E^ (Cthe_0446) for the late-log time point, and decreased expression of σ^K^ (Cthe_1012) for both time points in the standard medium comparison (Table 1). The PM has increased expression of σ^E^ (Cthe_0447) and σ^F^ (Cthe_0120) for the mid-log time point and decreased expression of σ^K^ (Cthe_1012) for both time points in the hydrolysate medium comparison (Table 1). A recent study of *C. acetobutylicum* showed that σ^K^ is involved in both early and late sporulation [40]. In *C. acetobutylicum sigK* deletion blocks sporulation, prior to Spo0A expression and the mutant suffered from premature cell death due to excessive medium acidification in batch cultures without pH control [40]. The sigK defective mutant did not transition into stationary phase where cells re-assimilate the acids and produce acetone, butanol, and ethanol [40]. The results suggest a positive-feedback loop between Spo0A and σ^K^ which may be the mechanism that down regulates Cthe_0812 for the PM in standard medium compared to the WT [40].

Sporulation is an energy intensive function requiring transcription of a large number of genes. By reducing the expression of certain sporulation genes, the PM may be capable of devoting more resources to growth. Furthermore, it has been shown that *C. thermocellum* forms L-forms upon depletion of substrate [35]. It is possible the PM favors L-forms over sporulation as a mechanism to conserve energy and promote faster recovery [35]. Once the genes that control the transition to L-forms have been discovered, this hypothesis can be tested.

Microorganisms are faced with the constant threat of invading foreign DNA, by genetic elements such as phages, plasmids, transposons and genomic islands [41]. However, in controlled environments such as the laboratory conditions used during directed evolution of this strain, these defense mechanisms may play a less important role in survival. Of the genes which encode for various cell defense mechanisms, the PM downregulated the expression of 29 and 46 genes compared to the WT in standard and *Populus* hydrolysate media, respectively. There are three subgroups of genes that represent the majority of the cellular defense genes: CRISPR associated proteins, Hedgehog/intein hint domain proteins and phage related proteins. Together these three subgroups make up 65 of the 94 cellular defense genes (Additional file 5). Odds ratios conducted on each of the three subsets of genes indicated that the difference of expression for each sub- group was statistically significant for both standard and *Populus* hydrolysate media comparisons. Although, defense mechanisms have their advantages, the PM may reduce the expression of the CRISPR-associated genes and Hedgehog/ intein hint domain protein in an effort to conserve cellular resources. Since the PM did not delete the CRISPR-associated regions, it still has the ability to recognize the foreign DNA. However, the reduced expression of these two groups of genes may come at the expense of increased expression of phage associated genes. *C. thermocellum* has 34 genes which encode for various phage-associated proteins which are not typically considered part of the cell defense mechanisms. The PM has an average 2-fold increased expression of 6 phage associated genes compared to the WT in standard medium which was deemed significant by the odds ratio. Conversely, the PM has an average 4-fold decreased expression of 16 phage associated genes compared to the WT in *Populus* hydrolysate medium which was also deemed significant by the odds ratio. The change in expression may be due to the increase in the expression of phage genes in the WT standard versus *Populus* hydrolysate media comparison below.

*C. thermocellum’s* rapid growth on crystalline cellulose is facilitated by a membrane bound complex, termed the cellulosome which consists of cellulases and other polysaccharide degrading enzymes assembled together in large protein complex [12],[42]. The primary scaffoldin protein of the cellulosome complex is attached to the cell wall and binds various carbohydrate degrading enzymes [12]. Cells are tightly attached to insoluble substrates via the carbohydrate binding module (CBM) often located at the distal end of the cellulosome complex [12]. However, the composition of carbohydrate active enzymes of the cellulosome differs as a function of the growth conditions [43]. The PM has a decreased expression of 19 and 42 of the 99 genes that encode for cellulosomal components in standard and *Populus* hydrolysate media, respectively (Additional file 4). The statistically significant decreased expression in cellulosome genes by the PM may be an attempt to conserve energy since the cells were adapted in media containing cellobiose and soluble glucans present from the hydrolysate. It has been hypothesized that the downregulation of the cellulosome on soluble substrate such as cellobiose occurs via catabolite repression [42]. The PM has a synonymous SNP at codon 415 in *RsgI6* (Cthe_2119) which is an anti-σ^I^ factor involved in regulating the expression of cellulosomal genes in the presence of xylans and cellulose [17]. It is possible that this mutation changes the specificity of the anti-σ^I^ factor and reduces the expression of the cellulosomal genes over and above the reduction that would be achieved by catabolite repression alone.

The PM has lower expression than the WT of 31 and 54 genes that encode for cell envelope proteins in standard and *Populus* hydrolysate medium (Additional file 4). The PM also downregulated 21 and 50 genes that encode for cell motility in standard and *Populus* hydrolysate media compared to the WT. It has been proposed that the σ^D^ in *B. subtilis* controls flagellin production and possibly has a role in the expression of the methyl-accepting chemotaxis proteins [31]. Sigma factor σ^D^ (Cthe_0495) is downregulated in the PM compared to the WT in standard and *Populus* hydrolysate media by 3-fold and 10-fold at the mid-log time point (Table 1) and may cause the decrease in cell motility genes. The PM also downregulated 12 genes that encode for various inorganic ion transport and metabolism proteins compared to the WT in standard medium and upregulates 17 genes in 10% v/v *Populus* hydrolysate medium. However, the downregulated genes do not belong to any specific pathway. The change in expression may be due to the downregulation of inorganic ion transport and metabolism genes in the standard versus *Populus* hydrolysate media comparison below. The PM also downregulated 26 genes in the miscellaneous category compared to the WT in standard medium. Beyond a simple conservation of cellular resources, the benefits of reducing the expression level of genes in these categories are unclear.

### Hydrolysate comparison

The *Populus* hydrolysate concentration comparison represents the difference in gene expression for various hydrolysate concentrations within a given strain. Inhibitory compounds from the *Populus* hydrolysate may affect the cell by damaging and denaturing biological molecules, resulting in adverse outcomes, including the improper folding of proteins, DNA damage, improper RNA folding and degradation, and the impairment of biophysical changes to cell membranes necessary for energy generation and the proper functioning of molecular pumps [44],[45]. The relatively small number of differentially expressed genes (total of 92 genes) for the PM in 10% v/v *Populus* hydrolysate compared to standard medium indicates that the PM strain requires relatively few changes in gene expression to adapt to the hydrolysate medium (Figure 1). This is not entirely surprising given that the PM was adapted to the hydrolysate during the directed evolution process. Even when the PM strain is placed in 17.5% v/v *Populus* hydrolysate, significant changes in expression occur in a total of 489 genes, compared to 1040 genes for the WT in 10% v/v *Populus* hydrolysate (Figure 1). All of the differentially expressed genes are listed in Additional file 4. The symmetry between induced and repressed genes in the standard versus hydrolysate conditions (Figure 1) suggests that a global conservation principle, possibly imposed by finite cellular resources, is involved in the dynamics of the genetic regulatory system [46]. Analysis of the categories with a significant number of differentially expressed genes may provide insight into the differences in these two strains. In response to hydrolysate, the PM upregulates genes related to growth and downregulates genes related to adaptation or survival, whereas the WT upregulates genes related to survival and downregulates growth genes. In summary, the hydrolysate initiates a stress-link response in the WT, but not in the PM. Only one category of genes is similarly regulated between the two strains.

#### Upregulated genes in the PM in hydrolysate media

The genes that are significantly upregulated by the PM in hydrolysate conditions belong to energy production and conversion, amino acid transport and metabolism, inorganic ion transport and metabolism, and general transport and secretion (Figure 1).

The PM increased the expression of five energy production and conversion genes in 10% v/v *Populus* hydrolysate, which represents a significant increase in expression within this category as determined by the odds ratio. The PM also increased the expression of 12 genes in this category in 17.5% v/v *Populus* hydrolysate; however, this increase was not significant due to the larger overall number of changes in gene expression. Specific differentially expressed genes related to the central metabolism can be seen in Table 3. Similarly, *C. acetobutylicum* upregulated genes related to energy production and metabolism in acetate and butyrate stress [13]. An NADPH-dependant alcohol dehydrogenase (ADH6p) was identified as one of the enzymes responsible for HMF and furfural reduction in *S. cerevisiae.* Furthermore, mutants with gene deletions along the pentose phosphate pathway (PPP) exhibited growth deficiency in the presence of furfural indicating that *S. cerevisiae* tolerance to furfural was associated with the activity of PPP. The increased expression in PPP genes in the PM strain in hydrolysate might assist in protecting against and repairing furfural induced damage [47].

The expression levels of the amino acid transport and metabolism genes do not change expression levels for the PM in the hydrolysate conditions. Since the PM upregulated these genes in standard medium compared to the WT, this means that the amino acid transport and metabolism genes remain elevated in the hydrolysate conditions. Conversely, *C. acetobutylicum* had a relatively large number of up- and down- regulated amino acid transport and metabolism related genes in acetate, butyrate and butanol stress [13]. The significantly upregulated histidine metabolism remains elevated in the hydrolysate condition with the exception of one gene Cthe_3028 which is down regulated. Histidine may be limited under furfural conditions so the further reduction of Cthe_3028 stops the conversion of histidine into histamine. The two terminal steps in histidine biosynthesis involve the reduction of NAD^+^ to NADH, a reaction that may be slowed by the high NADH/NAD^+^ ratio associated with fermentation [33]. Histidine has been shown to contribute to acid tolerance and *C. acetobutylicum* increases the expression of the histidine biosynthesis pathway when exposed to butanol and butyrate stress [13],[48].

The patterns of sulfur transport and metabolism of the WT in response to hydrolysate are complex. The PM upregulated 3 genes belonging to inorganic ion transport and metabolism in 10% v/v *Populus* hydrolysate compared to standard medium. In 17.5% v/v *Populus* hydrolysate a total of 18 genes experienced significant changes in regulation, including both up- and down-regulation. For the PM in 17.5% v/v *Populus* hydrolysate, four of the upregulated genes belonged to the sulfate ABC transporter, while 4 downregulated genes belonged to the phosphate ABC transporters. This suggests an increase in sulfur metabolism within the PM cell. In addition, of the 27 genes in the cysteine and methionine metabolism pathway, 3 were upregulated in the PM in 10% v/v *Populus* hydrolysate and 6 were upregulated in 17.5% v/v *Populus* hydrolysate; both changes are significant with respect to the odds ratio (Table 5). Up regulated genes include two copies of the *metY* gene (Cthe_1569 and Cthe_1842) which converts serine and hydrogen sulfide into L-cysteine and Cthe_1560 and Cthe_1840 which function along the same pathway. Together, upregulation of genes related to inorganic sulfur transport and cysteine synthesis are consistent with an attempt by the cell to overcome the detrimental effects of furfural on sulfate assimilation [13],[14],[33]. However, the sulfate reduction pathway is not observed to be upregulated. It is noteworthy that both copies of the *metY* gene underwent mutations late in the directed evolution process that would seem to inactivate them [17]. Cthe_1569 has a stop codon inserted at amino acid 229 and Cthe_1842 has a non-synonymous SNP (P29Q) in a highly conserved region [17]. With the disruption of the cysteine synthesis pathway, cells could still obtain cysteine directly from the medium. It is possible that the mutations which resulted in the upregulation of the sulfate uptake and cysteine synthesis pathways occurred earlier in the directed evolution process and were made superfluous by the late-occurring mutations in the *metY* genes.

**Table 5:** Fold change in gene expression along the cysteine and methionine metabolic pathway

**Gene**	**Product**	**PM vs. WT 0**		**PM vs. WT 10**		**PM 0 vs. 10**		**PM 0 vs. 17.5**		**WT 0 vs. 10**	
		**ML**	**LL**	**ML**	**LL**	**ML**	**LL**	**ML**	**LL**	**ML**	**LL**
Cthe_0290	homoserine dehydrogenase	−1.03	1.21	**2.33**	1.94	−1.78	−1.38	1.35	1.17	1.45	−1.30
Cthe_0580	aminotransferase class I and II	1.22	1.48	−1.31	1.17	1.03	−1.00	1.44	**2.03**	1.64	1.26
Cthe_0715	S-adenosylmethionine decarboxylase proenzyme	1.21	1.33	**2.95**	−1.12	−1.51	−1.64	−1.87	**−2.76**	**−3.67**	−1.10
Cthe_0755	aminotransferase class I and II	−2.42	−1.28	1.59	−1.40	−1.75	−1.37	−1.60	−2.06	**−6.77**	−1.25
Cthe_0961	aspartate-semialdehyde dehydrogenase	**−2.51**	**−2.11**	**−2.12**	−1.37	1.18	1.15	1.47	**2.34**	−1.01	−1.34
Cthe_1053	L-lactate dehydrogenase	−1.78	−1.25	1.32	−1.02	−1.41	−1.27	−1.33	−1.16	**−3.30**	−1.55
Cthe_1200	Adenosylhomocysteinase	−1.26	1.07	**2.23**	1.76	1.39	1.18	1.01	−1.62	**−2.02**	−1.39
Cthe_1559	Cys/Met metabolism pyridoxal-phosphate-dependent protein	**−9.22**	**−5.72**	**−4.73**	**−3.97**	**4.66**	**3.12**	**16.05**	**8.31**	2.39	2.17
Cthe_1560	Pyridoxal-5'-phosphate-dependent protein beta subunit	**−6.16**	**−2.97**	**−3.71**	**−2.65**	**6.25**	**3.41**	**15.55**	**6.43**	**3.77**	**3.05**
Cthe_1569	Cys/Met metabolism pyridoxal-phosphate-dependent protein	1.02	1.09	**−2.06**	**−1.83**	**3.94**	**2.46**	**5.21**	**4.42**	**8.24**	**4.90**
Cthe_1728	DNA-cytosine methyltransferase	**2.09**	**2.38**	**−1.21**	**2.26**	1.03	−1.01	1.59	1.80	**2.60**	1.04
Cthe_1749	DNA-cytosine methyltransferase	1.08	−1.12	**−5.98**	**−2.41**	−1.08	1.20	1.13	1.46	**5.95**	**2.58**
Cthe_1840	cysteine synthase A	−1.52	−1.21	**3.14**	**2.17**	1.37	1.27	1.83	−1.27	**−3.48**	**−2.07**
Cthe_1842	O-acetylhomoserine/O-acetylserine sulfhydrylase	−1.68	−1.54	−1.10	1.52	1.51	1.16	**2.46**	1.75	−1.02	−2.01

Bold values indicate significantly different levels of express as determined by ANOVA. For the PM vs. WT in 0% and 10% v/v *Populus* hydrolysate, a positive/negative value represents a higher/lower expression level in the PM compared to the WT. For the standard medium (0%) versus *Populus* hydrolysate media (10 or 17.5%) positive/negative values represents higher/lower expression levels in the hydrolysate media compared to standard medium. Values are indicated for samples collected during mid-log (ML) and late-log (LL) growth phases.

The genes that belong to the general transport category are basic ABC transporter and glycosyl transferase groups which are labeled with multiple COG designations. ABC transporters utilize ATP energy to transport inorganic ions, amino acids, hydrocarbons, polypeptides or hydrophobic compounds [44]. In some Gram-positive organisms, the ATP-binding subunit of an ABC system is not part of a specific transporter complex; instead, it is shared by multiple transporters [49] increasing the efficiency of the cell. The PM in 17.5% v/v *Populus* hydrolysate upregulated 16 of the 143 genes that encode for various transport genes compared to standard medium. This may allow for faster transport of compounds into the cell or inhibitors out of the cell, allowing the faster growth phenotype (Additional file 4).

#### Downregulated genes in the PM in hydrolysate media

A change in the environment causes a response of the genetic network which in turn allows efficient plastic adaptation of cellular metabolism to a broad range of unforeseen challenges [46]. Increased transcriptional flexibility allows the cells to address challenges on physiological timescales (not through new mutations) [46]. The PM in 10% v/v *Populus* hydrolysate decreases the expression of 8 transcription genes, and in 17.5% v/v *Populus* hydrolysate it decreases the expression of 22 genes (Additional file 4). In addition the PM in 10% v/v *Populus* hydrolysate decreases the expression of four genes in the cell defense mechanism category which was determined significant by the odds ratio because of the small total number of genes being differentially expressed. Cell defense mechanisms and the ability to rapidly change its transcriptional profile in response to changing environments normally contribute to cell fitness; however, these traits may be less advantageous in a steadily-maintained, pure-culture laboratory environment. As a result, the PM may be decreasing expression of cell defense and transcriptional genes as an energy saving mechanism.

#### Upregulated genes in the WT in hydrolysate medium

The WT in hydrolysate medium significantly upregulates two categories of genes that relates to survival mechanisms: cell defense mechanisms and cell motility genes. The WT already had a higher expression of the cell defense mechanism genes compared to the PM in standard medium which is further increased in hydrolysate medium. In 10% v/v *Populus* hydrolysate the WT increased the expression of 38 cellular defense genes compared to standard conditions (Additional file 4). The WT has an average 2-fold higher expression of 8 genes that encode Hedgehog/intein hint domain proteins and 18 phage-associated proteins in hydrolysate medium compared to standard medium. These increases are possibly part of a programmed cell response to the general deterioration of the cell health in hydrolysate conditions. While these increases in gene expression environment may help the cell to survive in a natural environment, they drain resources away from central metabolism and ethanol production. The WT in 10% v/v *Populus* hydrolysate also increases the expression of 44 cell motility genes and upregulates the expression of sigma factor σ^D^ by 3-fold (Table 1). The increase in motility of the WT in response to hydrolysate may be an attempt by the cell to swim away from unfavorable environments (Additional file 4). In contrast, the PM may not see the hydrolysate conditions as an unfavorable environment and further conserves energy by reducing the expression of the cell motility genes. However, a transcriptional analysis of *Clostridium beijerinckii* found that the genes were downregulated during the switch from acidogenesis to solventogenesis during fermentation [11]. Furthermore, *C. acetobutylicum* also downregulates cell motility genes in acetate stress but increases the expression in butyrate stress [13].

#### Downregulated genes in the WT in hydrolysate

The WT in 10% v/v *Populus* hydrolysate medium downregulates the expression of the sigma factor σ^A^ gene Cthe_1809 by 2-fold compared to standard medium, which may contribute to the observed slower growth phenotype. Since the change in expression of Cthe_1809 is closely related to the observed growth rates in both the WT and PM, it may be one of the more important genes that encode for sigma factor σ^A^ in *C. thermocellum*. The WT in 10% v/v *Populus* hydrolysate does upregulate a sigma 70 region 2 domain protein; however, the protein is approximately half the length of the genes encoding for the RNA polymerase sigma factors; therefore, its exact function is unknown. Although, the WT in 10% v/v *Populus* hydrolysate does not decrease the overall expression of the energy production and conversion genes compared to standard medium, it does significantly down regulate the operon Cthe_0422-3. The wild type strain of *C. thermocellum* has shown a similar response where genes Cthe_0422-0432 were the most strongly downregulated upon exposure to furfural [14]. *C. acetobutylicum* also downregulates *rex,* a regulator of solventogenesis, under butyrate stress [48].

The WT in 10% v/v *Populus* hydrolysate decreases the expression of 37 genes in the cell envelope category compared to standard medium (Additional file 4). The WT also downregulated 11 of the 45 genes belonging to lipid degradation and biosynthesis in this comparison (Additional file 4). Organic solvents can damage the membrane structure and destabilize the function of its associated proteins [50]. Lipoprotiens are proposed to maintain the structure and function of bacterial cell envelopes [51]. *C. acetobutylicum* is inhibited by solvents which change the lipid composition and disrupts the cell membrane fluidity [50],[51]. Transcriptomic analysis of *C. acetobutylicum* found that genes with cell envelope associated functions were the largest group to be up- and down- regulated in butanol stress conditions; however, genes involved with lipid biosynthesis were upregulated [50],[51]. The reduction of cell envelope and lipid degradation and biosynthesis pathways suggests that the WT does not have the energy required to exert the elaborate and highly sophisticated regulation of these pathways in 10% v/v *Populus* hydrolysate[52].

The WT also downregulated a significant number of amino acid transport and metabolism genes (33 genes) in 10% v/v *Populus* hydrolysate compared to the standard medium (Additional file 4). However, the change in gene expression did not belong to a specific pathway. The WT downregulates 19 genes belonging to inorganic ion transport and metabolism in 10% v/v *Populus* hydrolysate compared to standard medium including all 8 ABC transporter genes that were increased in the PM hydrolysate comparisons (Additional file 4). This possibly reduces the amount of sulfate-derived sulfur and phosphate available in the cell. However, the fact that the WT could obtain cysteine directly from the media may have reduced its need to transport sulfate for synthesis of sulfur-containing amino acids, allowing more of the NADPH to be allocated to furfural oxidation [33].

#### Similarly expressed category

The PM in 17.5% v/v *Populus* hydrolysate increases the expression level of 14 genes encoding for the cellulosome. Similarly, the WT in 10% v/v *Populus* hydrolysate increases the expression level of 30 genes encoding for the cellulosome. The majority of the genes with increased expression belong to various glycoside hydrolase (GH) families. The various GH families encode for endo- and exoglucanases used to degrade the cellulose components [12],[42]. The PM in 17.5% v/v *Populus* hydrolysate increases the expression of 8 GH family proteins, and the WT in 10% v/v *Populus* hydrolysate increases the expression of 18 GH family proteins. *Populus* hydrolysate does not contain any solid cellulose or hemi-cellulose; however, it does contain significant amounts of other soluble sugars from the original pretreated biomass. The concentration of sugars in the full (100%) *Populus* hydrolysate include glucose (22.7 g/L), xylose (42.7 g/L), arabinose (1.84 g/L), and mannose (6.34 g/L) [17]. These molecules may play the role of signaling molecules in the regulation of cellulosomal gene activity, thereby accounting for the greater expression of cellulosomal genes in hydrolysate media [53].

## Conclusion

**Figure 4:**
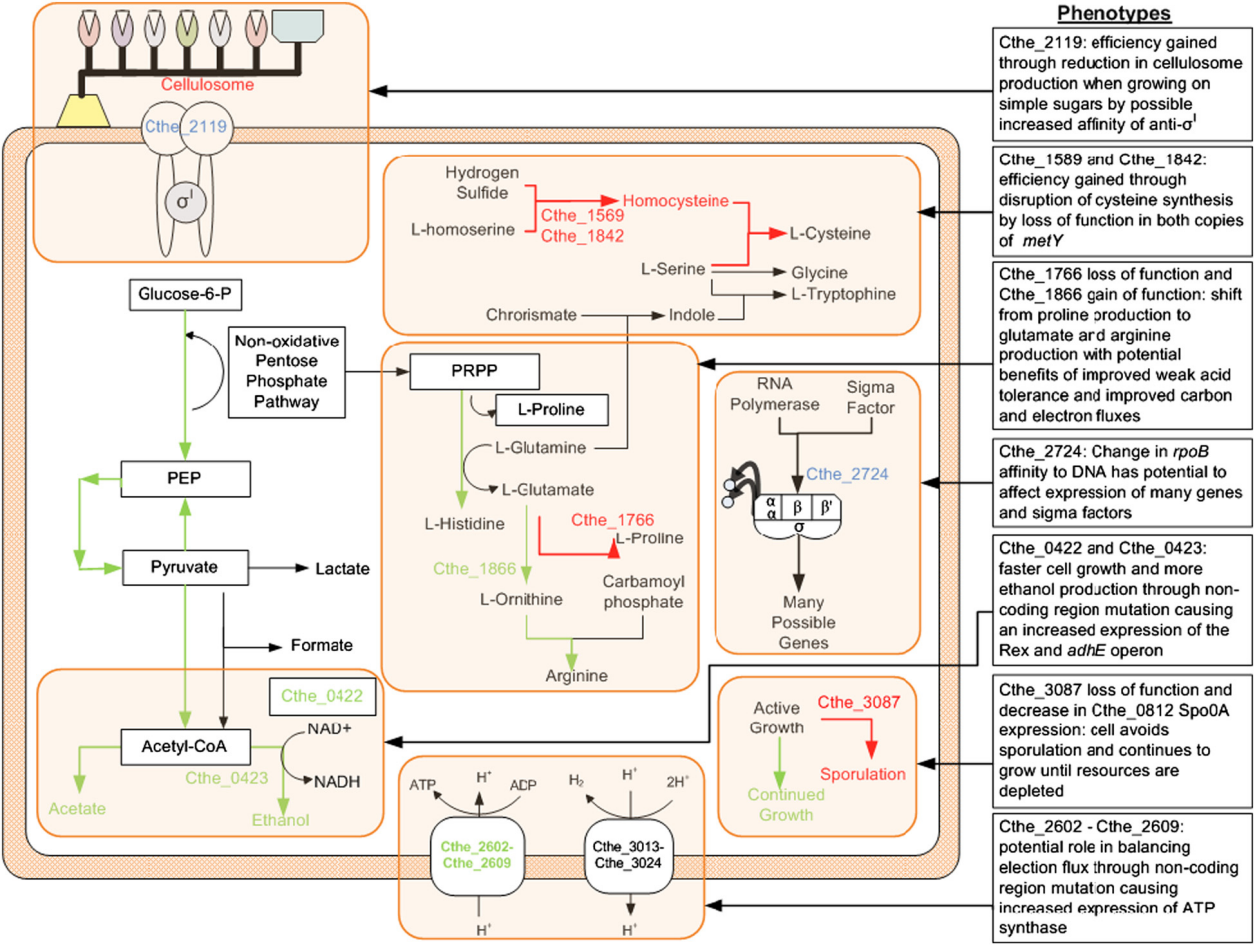
**Summary of mutations and resulting changes in gene expression and phenotypes in the PM.** Pathways (and related mutations in specific genes) with increased (green) or decreased (red) expression or functionality are shown. Mutations shown in blue do not lead to a change in gene expression but affect the affinity of the protein. The resulting phenotypic changes leading to hydrolysate tolerance are also shown.

A summary of the major mutations and related changes in gene expression or pathway activity and associated phenotypes that impart hydrolysate tolerance is shown in a conceptual model of the PM strain in Figure 4. No single mutation could explain the performance difference of the two strains; rather, several mutations each seem to impart small advantages that cumulatively contribute to the tolerance phenotype of the PM. Mutations contributed to diverted carbon and electron flows, interruption of the sporulation mechanism, modifications to the transcriptional machinery potentially leading to widespread changes in gene expression, and efficiencies related to decreases in cellulosome and cysteine synthesis as a result of the cell adapting to the laboratory growth conditions.

The greater number of genes with decreased expression versus increased expression for the PM compared to the WT in standard and *Populus* hydrolysate media suggests increased cellular efficiency in the PM strain of *C. thermocellum*. The PM increases expression in the energy production and conversion category and in the histidine biosynthesis pathway compared to the WT in standard medium. The PM also increased the expression of genes belonging to the inorganic ion transport and metabolism category compared to the WT in 10% v/v *Populus* hydrolysate. The PM has a decreased expression in a number of functional gene categories (sporulation (standard medium only), cell defense mechanisms, cell envelope biogenesis, cell motility, cellulosome, inorganic ion transport and metabolism (standard medium only) and miscellaneous genes (standard medium only)) allowing for greater efficiency. The high similarity in gene expression of the PM compared to the WT in both standard and *Populus* hydrolysate media may be due to the few changes in gene expression of the PM in the standard versus *Populus* hydrolysate media comparison. The PM strain grown in hydrolysate media versus standard medium showed fewer differentially expressed genes than the WT strain when grown in the same two conditions suggesting that there is a more targeted response to the *Populus* hydrolysate by the PM strain than the WT strain. The PM upregulates genes related to growth processes and downregulates genes related to survival mechanism in the hydrolysate conditions. The WT had the opposite response when placed in the hydrolysate medium. These expression level changes for the PM may be detrimental to survival in natural environments but allowed for the better growth in the laboratory environment in which the strain was evolved, thus likely allowing for better survival and bioconversion efficiency in future production facilities producing biofuels.

## Methods

### Strain and culture conditions

*C. thermocellum* ATCC 27405 was obtained from Prof. Herb Strobel, University of Kentucky collection and denoted as the wild type (WT) strain. A *Populus* hydrolysate-tolerant strain, referred to as the *Populus* Mutant (PM) strain was developed from the WT strain and has been previously described [17]. Media, *Populus* hydrolysate, and culture conditions, fermentation procedures, RNA extraction and isolation techniques, sequencing procedures, and RNA expression analysis were previously described [17]. The sequenced reads NCBI study accession number is SRP024324.

### RNA analysis

JMP Genomics Version 10 (SAS, Cary, NC) was used to analyze the gene expression data. Raw count data was log-2 transformed and normalized by the Upper Quartile Scaling method [54],[55]. Two samples were removed from subsequent analysis due to poor data quality. An analysis of variance (ANOVA) test was conducted on each independent variable and the three independent variables together in simple comparisons using a false discovery rate method of nominal α, p <0.05. For the simple comparisons, genes were considered significantly differently expressed at a log-2 difference greater than 1 (representing a 2-fold change in expression) and a –log10(p) was greater than 2.126. Further analysis was conducted based on an expanded version of Clusters-of-Orthologous groups (COGs) [12],[56]. The new annotation of *C. thermocellum* lists the JGI categorizations which do not correspond directly to COG categories. ORNL computational biology group has also defined COG categories for 1928 genes in the new annotation of *C. thermocellum*. Both can be found here: http://genome.ornl.gov/microbial/cthe/[55]. Additional categories were assigned for subcategories of COGs such as cellulosomal genes and transport and secretion genes. Genes were initially assigned to COGs during the annotation using RPS Blast and refined via manual curation as shown in (Additional file 1: Table S2). The full list of genes with category definition can be found in Additional file 5. To determine the significance of up or down regulation within a given category, an odds ratio of the number of up- or down-regulated genes in a category versus the total number of up- or down- regulated genes across the genome was used with a normally distributed 95% confidence interval (α = 0.05). Odds ratios of certain additional subsets of genes were conducted to further determine significance [57].

### Quantitative-PCR (qPCR) analysis

RNA-seq data were validated using real-time qPCR, as described previously [7],[8], except that the Bio-Rad MyiQ2 Two-Color Real-Time PCR Detection System (Bio-Red Laboratories, CA) and Roche FastStart SYBR Green Master (Roche Applied Science, IN) were used for this experiment. Six genes were analyzed using qPCR from cDNA derived from the mid-log time point samples for the WT and PM in standard media.

## Competing interests

CDC has a financial interest (stock ownership) in Renmatix, Inc. Renmatix is developing technology to produce sugars from biomass via abiotic processes. He acquired stock by exercising options awarded to him as compensation for providing technical advice in the early history of the company. He no longer has any relationship with the company other than stock ownership. It is unlikely that he would be able to affect the future value of the stock through this publication, even if he were motivated to do so. CDC is the Director of the Institute for a Secure and Sustainable Environment which provided funding to support JLL through institutional funds that he has been entrusted to administer. This does not alter our adherence to all the *BMC Microbiology* policies on sharing data and materials.

## Authors’ contributions

JLL conceived of the study, participated in the design of experiments, performed all experiments, analyzed and interpreted data. MR conceived of the study, participated in the design of experiments and contributed to the fermentation experiments. SDB conceived of the study, participated in the design of experiments, contributed to the analysis and interpretation of the data. JRM conceived of the study, participated in the design of experiments, contributed to the analysis and interpretation of the data. CDC conceived of the study, participated in the design of experiments, contributed to the analysis and interpretation of the data. All authors read and approved the final manuscript.

## Additional files

## Supplementary Material

Additional file 1 **Supplemental Information.** Contains all supplementary tables and figures. Click here for file

Additional file 2 **All statistically significant differentially expressed genes.** Contains an Excel file with all of the results of the ANOVA test for all 1,795 statistically significant differentially expressed genes based on all possible simple comparisons. Click here for file

Additional file 3 **Significantly differentially expressed hypothetical proteins.** Contains an Excel file with the 551 genes that encode hypothetical proteins, pseudo genes, and genes of unknown function. Click here for file

Additional file 4 **Significantly differentially expressed genes with category designation.** Contains an Excel file with the 1189 genes that were significantly differentially expressed along with the category designation assigned by this analysis. Click here for file

Additional file 5 **Genes and category definitions.** Contains an Excel file with one tab describing how the 20 categories define in this manuscript relate to JGI color categories and COGs. The other tab lists the 2,312 genes with known function that was placed into one of the 20 categories. Click here for file
