# Supplementary material for: Transcriptomic analysis of Clostridium thermocellum Populus hydrolysate-tolerant mutant strain shows increased cellular efficiency in response to Populus hydrolysate compared to the wild type strain
Source: BMC Microbiol. 2014 Aug 16;14:215. doi: 10.1186/s12866-014-0215-5 (PMC4236516; doi:10.1186/s12866-014-0215-5)
Supplement: Additional file 1 — Supplemental Information. Contains all supplementary tables and figures. [file s12866-014-0215-5-S1.docx]

**Additional File 1: Supplemental Information.**

**Figure S1. Growth comparison for the WT and PM strains of *C. thermocellum* in batch fermentations with different concentration of *Populus* hydrolysate containing media.** A) Dry Cell weight (g/L), B) Sugar utilization measured as glucan (g/L) which is cellobiose + glucose, C) ethanol production (g/L) and D) adjusted acetic acid production (g/L). The 10% v/v *Populus* hydrolysate medium contain 1.3 g/L acetic acid and the 17.5% v/v *Populus* hydrolysate medium contained 2.4 g/L acetic aicd which was subtracted from the total concentration. These figures adapted in part from [17, 18].

**
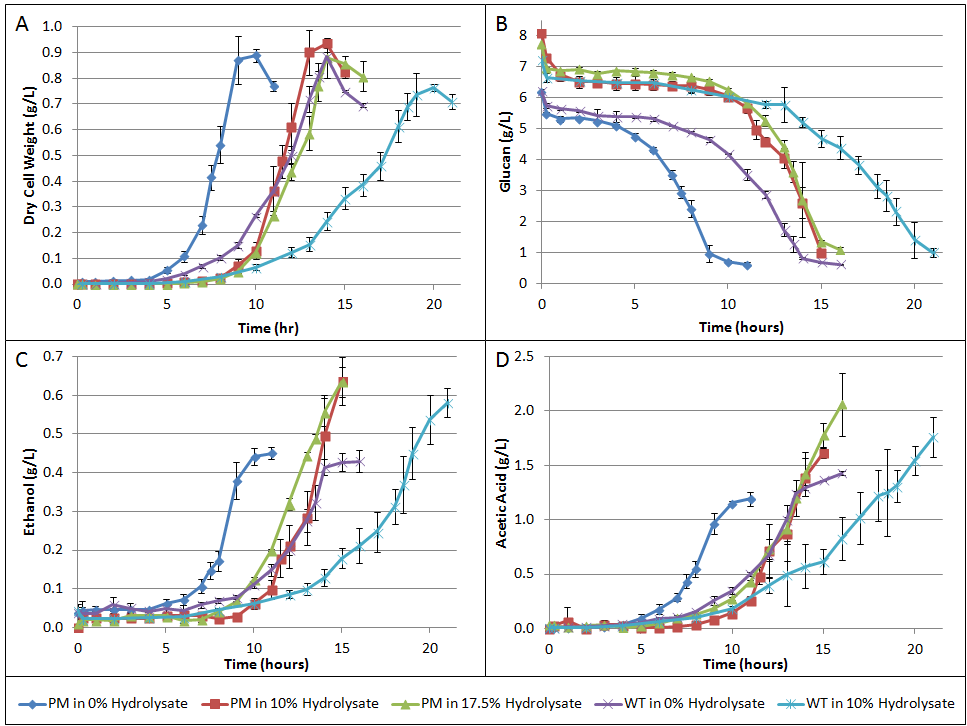
**

**Figure S2. Correlation between RNA-seq and real-time qRT-PCR results for RNA-seq data validation.** Samples were collected from growth cultures of *C. thermocellum* WT and PM in 0% v/v Populus hydrolysate at the mid-log time point. The gene expression ratios of both the RAseq data and qPCR data were log transformed in base 2 (log_2_<PM/WT>), and the expressionn ratios determined by the two methods compared. The six genes are Cthe_0191, Cthe_0508, Cthe_1028, Cthe_1822, Cthe_2186, and Cthe_2531.

**Table S1. Fermentation data for RNA-seq samples.** Samples were taken at either the mid-log or late-log time points based on growth rate. The table below shows the time point and average dry cell weight (DCW) for the triplicate fermentation samples.

|  | Mid-Log | | Late-Log | |
| --- | --- | --- | --- | --- |
|  | Time (hr) | DCW (g/L) | Time (hr) | DCW (g/L) |
| PM 0% | 7.5 | 0.415 + 0.10 | 9 | 0.872 + 0.09 |
| PM 10% | 11.5 | 0.364 + 0.10 | 13 | 0.612 + 0.09 |
| PM 17.5 | 12 | 0.438 + 0.03 | 13.5 | 0.771 + 0.07 |
| WT 0% | 12 | 0.502 + 0.03 | 13.5 | 0.810 + 0.05 |
| WT 10% | 15 | 0.335 + 0.04 | 18.5 | 0.690 + 0.05 |

**Table S2. Number of genes by category.** The table lists the category, category number used in supplementary files, total number of *C. thermocellum* genes in that category and the number of differentially expressed genes for that category in each of the four analyses performed. A positive (+) expression indicates that the gene was upregulated and negative (-) expression indicates the gene was downregulated by the previously described comparisons. Bold values indicate the number is of differentially expressed genes are significant by odds ratio. The number of hypothetical genes removed from the analysis and the total number differentially expressed genes as seen in Figure 1 are also included in the table.

| Category | Number | Number of Genes | PM vs. WT in 0% hydrolysate | | PM vs. WT in 10% hydrolysate | | PM  0 vs. 10% hydrolysate | | PM  0 vs. 17.5% hydrolysate | | WT  0 vs. 10% hydrolysate | |
| --- | --- | --- | --- | --- | --- | --- | --- | --- | --- | --- | --- | --- |
| **Gene Expression and Protein Production** |  |  | (+) | (-) | (+) | (-) | (-) | (+) | (-) | (+) | (-) | (+) |
| Transcription | 1 | 161 | 12 | 9 | 20 | 40 | **8** | 0 | **22** | 11 | 20 | 28 |
| Translation, Ribosomal Structure and Biogenesis | 2 | 185 | 9 | 5 | 25 | 18 | 1 | 1 | 15 | 7 | 20 | 37 |
| Post-translational Modification, Protein Turnover and Chaperones | 3 | 70 | 7 | 7 | 12 | 16 | 0 | 0 | 2 | 2 | 10 | 11 |
| **Cellular Growth Processes** |  |  |  |  |  |  |  |  |  |  |  |  |
| Cell Division and Sporulation | 4 | 116 | 0 | **20** | 10 | 23 | 0 | 1 | 12 | 7 | 20 | 9 |
| DNA Replication, Recombination and Repair | 5 | 255 | 12 | 12 | 22 | 36 | 4 | 5 | 13 | 22 | 16 | 31 |
| Cell Defense Mechanisms | 6 | 94 | 9 | **29** | 4 | **46** | **4** | 0 | 8 | 6 | 9 | **38** |
| **Cell Wall** |  |  |  |  |  |  |  |  |  |  |  |  |
| Cell Envelope Biogenesis | 7 | 179 | 1 | **31** | **44** | 18 | 3 | 0 | 10 | 9 | **37** | 26 |
| Signal Transduction Mechanisms | 8 | 79 | 4 | 9 | 12 | 18 | 0 | 0 | 6 | 4 | 10 | 7 |
| Cell motility | 9 | 106 | 6 | **21** | 3 | **50** | 2 | 0 | 4 | 9 | 7 | **44** |
| Cellulosome | 10 | 99 | 4 | **19** | 5 | **42** | 0 | 0 | 7 | **14** | 4 | **30** |
| **Metabolism and Transport** |  |  |  |  |  |  |  |  |  |  |  |  |
| Energy Production and Conversion | 11 | 152 | **23** | 4 | **44** | 18 | 1 | **5** | 10 | 12 | 20 | 20 |
| Carbohydrate Transport and Metabolism | 12 | 83 | 7 | 11 | 15 | 13 | 0 | 0 | 7 | 2 | 17 | 8 |
| Amino Acid Transport and Metabolism | 13 | 163 | **20** | 12 | **37** | 20 | 2 | 3 | 13 | 14 | **33** | 17 |
| Inorganic Ion Transport and Metabolism | 14 | 60 | 2 | **12** | **17** | 8 | 2 | **3** | **9** | **9** | **19** | 4 |
| Coenzyme Metabolism | 15 | 88 | 3 | 8 | 13 | 7 | 0 | 1 | 2 | 8 | 12 | 4 |
| Nucleotide Transport and Metabolism | 16 | 63 | 1 | 0 | 8 | 2 | 0 | 0 | 3 | 2 | 8 | 12 |
| Lipid Metabolism | 17 | 45 | 1 | 5 | 6 | 4 | 1 | 1 | 4 | 2 | **11** | 0 |
| Secondary Metabolites biosynthesis Transport and Catabolism | 18 | 20 | 0 | 0 | 1 | 1 | 0 | 0 | 1 | 1 | 2 | 0 |
| General Transport and Secretion | 19 | 143 | 11 | 13 | 15 | 31 | 1 | 6 | 11 | **16** | 17 | 30 |
| **Miscellaneous** |  |  |  |  |  |  |  |  |  |  |  |  |
| Miscellaneous | 20 | 151 | 7 | **26** | 18 | 38 | 1 | 2 | 12 | 4 | 27 | 25 |
| **Totals for odds ratio** |  | **2312** | **139** | **253** | **302** | **485** | **30** | **28** | **171** | **161** | **319** | **381** |
| Hypothetical |  | 975 | 47 | 140 | 69 | 295 | 23 | 11 | 89 | 68 | 128 | 212 |
| **Total** |  | **3287** | **186** | **393** | **371** | **780** | **53** | **39** | **260** | **229** | **447** | **593** |

**Table S3. Sub-categorization of the cell division genes for the PM vs. WT in standard and hydrolysate media comparison.** Subdivision of category 4: Cell Division and Sporulation genes show that only the sporulation genes have a significant change in expression by the odds ratio. Bold values indicate the number is of differentially expressed genes are significant by odds ratio.

|  |  | PM vs. WT in 0 | | PM vs. WT in 10 | |
| --- | --- | --- | --- | --- | --- |
|  | # of genes | upregulated | downregulated | upregulated | downregulated |
| Both Cell Division and Sporulation | 116 | 0 | **20** | 10 | 23 |
| Cell Division only | 33 | 0 | 3 | 2 | 6 |
| Sporulation only | 83 | 0 | **17** | 8 | 17 |
